# Hypertrophic obstructive cardiomyopathy with left ventricle outflow tract chordae insertion: Surgery or alcohol septal ablation? A case report

**DOI:** 10.1002/ccr3.7928

**Published:** 2023-09-21

**Authors:** Rania Hammami, Amine Kammoun, Majed Hassine, Tarek Ellouze, Rania Gargouri, Leila Abid

**Affiliations:** ^1^ Cardiology Department, Faculty of Medicine University of Sfax Sfax Tunisia; ^2^ Cardiology A Department Fattouma Bourguiba University Hospital, Cardiothrombosis Research Laboratory (LR12SP16) University of Monastir Monastir Tunisia

**Keywords:** alcohol septal ablation, anomalous chordae insertion, case report, hypertrophic obstructive cardiomyopathy

## Abstract

Anomalous insertion of chordae is a rare disease that could be associated with hypertrophic obstructive cardiomyopathy (HOCM), but clinical and echocardiographic diagnoses tend to be delayed. Alcohol septal ablation has emerged as an alternative to surgical myomectomy in HOCM. When a patient showed an anomalous insertion of chordae, physicians generally opt for surgery and not alcohol septal ablation. In this report, we present the case of a lady, with symptomatic HOCM associated with a chord inserted on the left ventricular outflow tract. We succeeded to relieve obstruction by alcohol septal ablation without the need for surgery.

## Graphical abstract

Abnormal insertion of mitral chordae associated with hypertrophic obstructive cardiomyopathy with significant turbulence across the LV outflow tract.

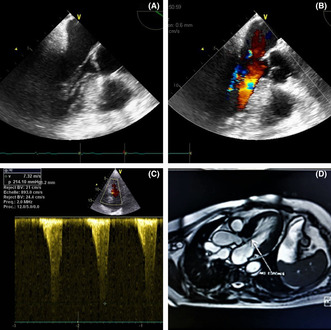

## 1 INTRODUCTION

Mitral subvalvular apparatus malformation is a group of rare congenital mitral malformations underdiagnosed due to low incidence.^1^ This congenital anomaly can be associated with hypertrophic cardiomyopathy (HCM) and can contribute to left ventricular outflow tract (LVOT) obstruction, beyond the systolic anterior motion of the anterior mitral leaflet.^2^ We report the case of hypertrophic obstructive cardiomyopathy (HOCM) with anomalous insertion of chordae on LVOT diagnosed by transthoracic echocardiography and cardiac MRI and successfully treated with septal alcohol ablation.

## 2 CASE PRESENTATION

A 62‐year‐old woman presented to the emergency with dyspnea NYHA II and lipothymia. Her medical history was unremarkable.

The cardiac auscultation showed an ejection systolic murmur (grade 4/6) in the aortic area. General examination and other systems examination were unremarkable. A 12‐lead electrocardiogram showed sinus rhythm at 90 beats/min with left ventricle hypertrophy.

A transthoracic echocardiogram (TTE) demonstrated an asymmetric interventricular septal hypertrophy of LV walls (septum = 20 mm, posterior wall = 14 mm at end‐diastole) with a normal ejection fraction but a severe subvalvular aortic stenosis with significant turbulence across the LV outflow tract (LVOT) in color Doppler and a late‐peaking “dagger‐shaped” systolic jet assessed by continuous‐wave Doppler.

The peak velocity exceeds 7 m/s with a maximal gradient of 210 mm Hg.

A transesophageal echocardiogram showed also a moderate systolic anterior motion with septal contact lasting less than a third of the systolic period and two chordae attached to the interventricular septum and mitro‐aortic trigona.

The aortic valve was tricuspid with a normal orifice area.

**FIGURE 1:**
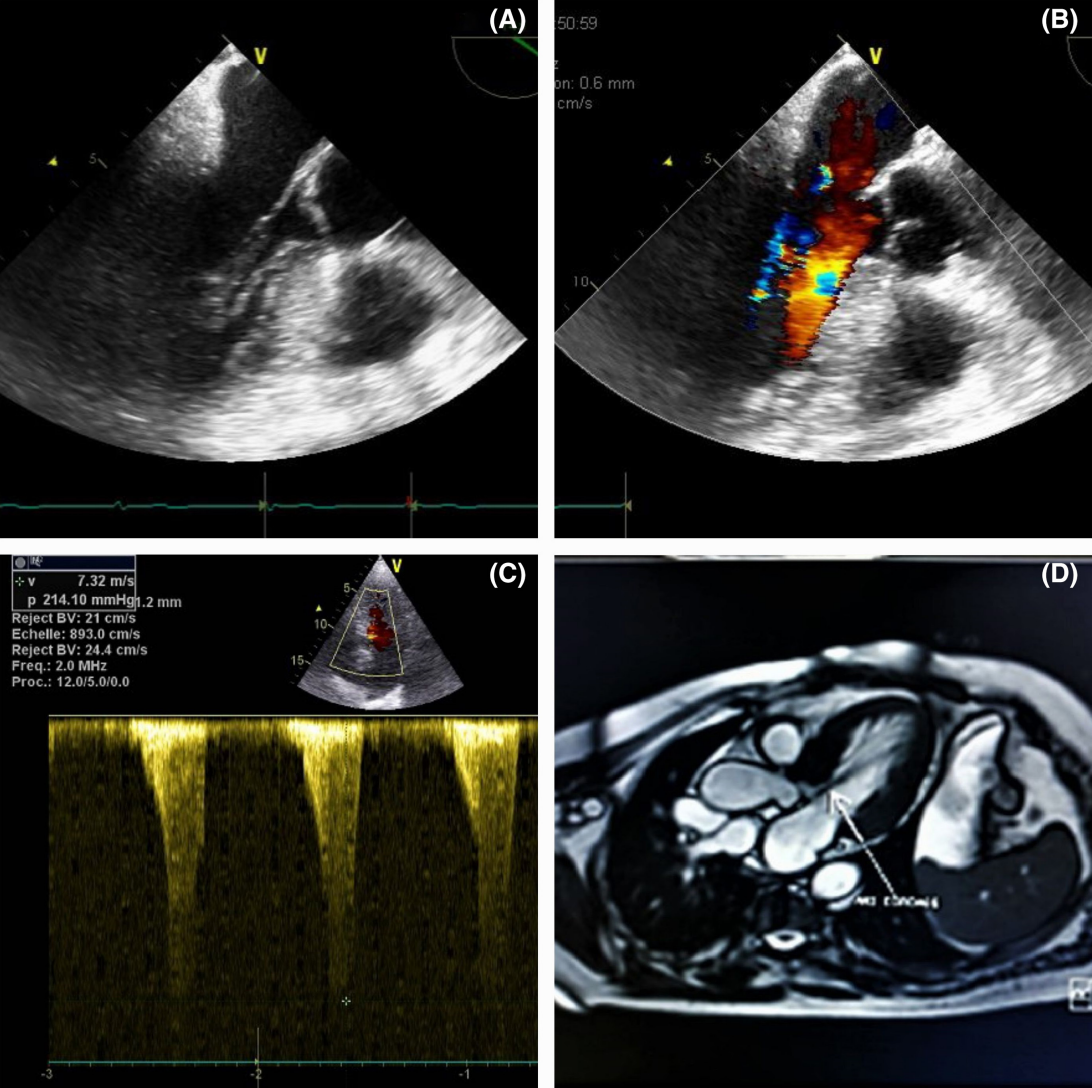
Imaging findings. (A) Apical view shows a significant LVOT narrowing. The septal wall showed increased thickness measured at 20 mm. Two subvalvular structures attached to the interventricular septum and the mitro‐aortic trigona were also visualized (blue arrows). (B) Color Doppler reveals a flow acceleration with aliasing across the LVOT (yellow arrow) with a moderate mitral regurgitation (orange arrow). (C) Continuous‐wave Doppler at the LVOT showing a peak velocity of 7.32 m/s and a maximal gradient ≈of 210 mm Hg. The waveform contour is dagger‐shaped. (D) MRI: Abnormal insertion of the chordae confirmed by MRI, obstruction which is more likely caused by septal hypertrophy than abnormal chordae.

MRI was also performed to evaluate the mitral subvalvular apparatus. It showed the two chordae crossing the left ventricular outflow tract, and the analysis of dynamic sequences showed that the obstruction is the consequence of the hypertrophy of the basal septal segment (Figure 1).

The evaluation of sudden cardiac death (SCD) using the HCM‐SCD score showed a score of 3%, and there was no indication of implanting a defibrillator.

The patient had been treated with beta‐blockers and calcium channel blockers for 3 months but remained symptomatic, and the LVOT obstruction did not improve.

The lady refused surgery and the heart team after discussion with the patient decided to try an alcohol ablation. The lady signed consent and accepted all the risks of the procedure.

Coronary angiography was performed via a transradial approach. The left main coronary artery was cannulated with an EBU 6 Fr guiding catheter, a 0.014 guidewire was placed into the proximal septal branch, a (2.5 × 10 mm) over‐the‐wire (OTW) balloon was advanced into the guidewire placing it in the ostium of the septal branch, and it was inflated up to 12 atm.

During probatory inflation of the balloon, the gradient resolved completely, and we were convinced that the obstruction is due to the septal hypertrophy, not to abnormal chordae. Desiccated alcohol of 96% was used.

**FIGURE 2:**
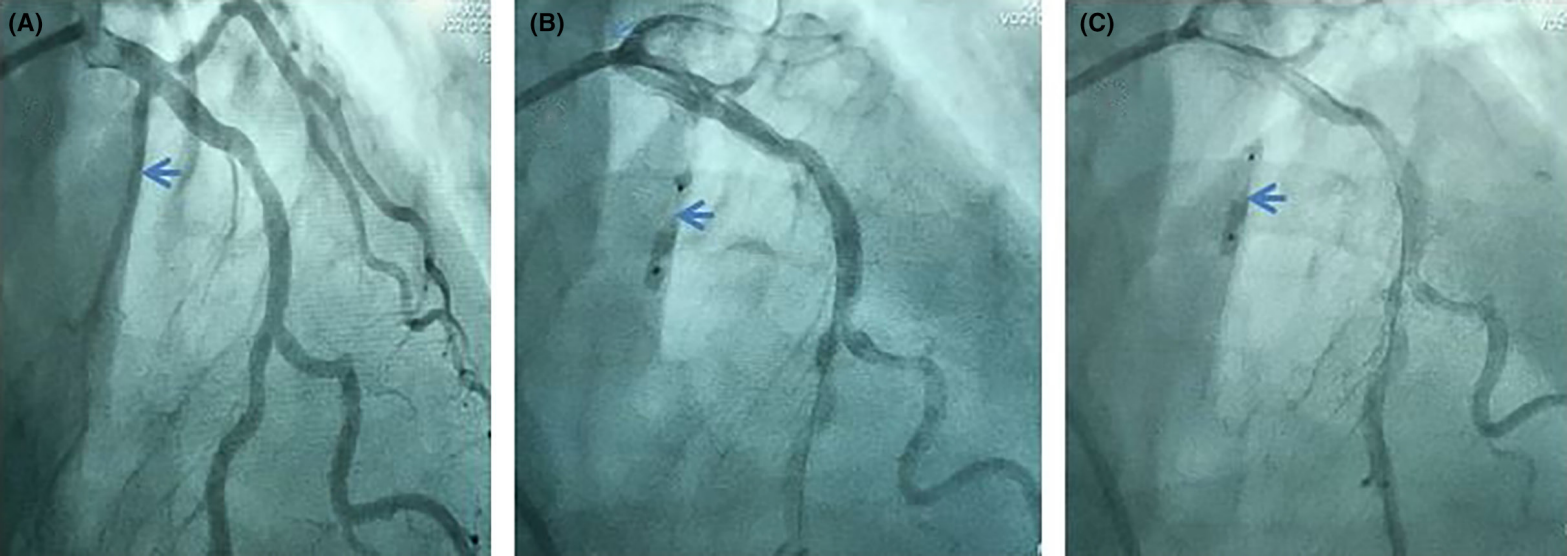
(A) Arrow pointing to the proximal septal branch of the left anterior descending artery. (B, C) An arrow pointing to the over‐the‐wire (OTW) balloon inflated during the alcohol injection.

Alcohol septal ablation (ASA) was performed with a single slow injection of 2 mL of alcohol over 4 min. We did not note any further complications. We obtained a slow flow in the first septal and the outflow tract gradient disappeared on invasive pressure measurements (Figure 2).

A peak creatine kinase (CK) after the procedure was at 1450 U/L.

**FIGURE 3:**
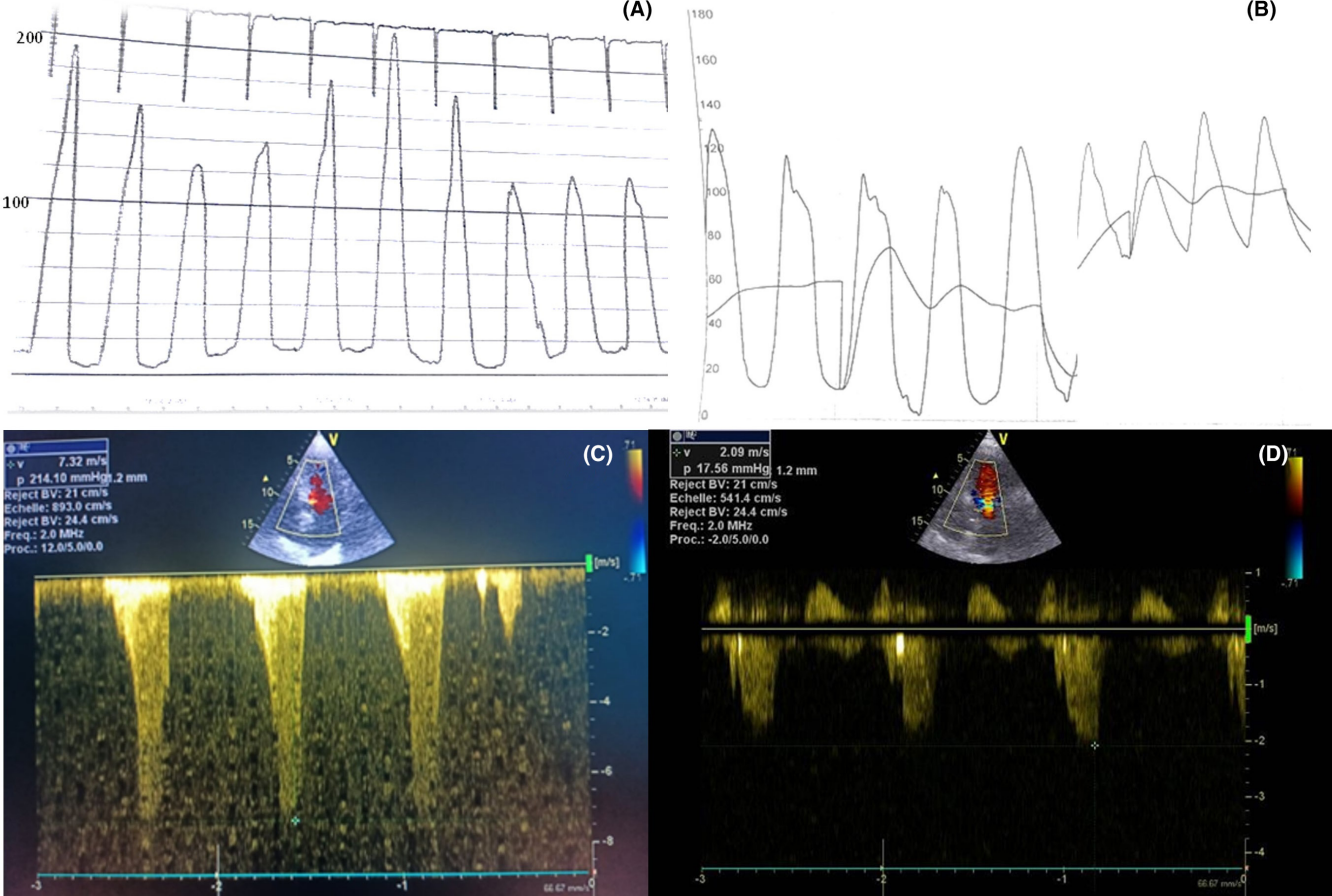
(A and C): Hemodynamic tracings at cardiac catheterization and TTE findings: At baseline, an LVOT gradient of 110 mm Hg was present, resulting from obstructive HCM (B and D): Hemodynamic tracings and TTE findings after successful alcohol septal ablation: the gradient immediately decreased.

**FIGURE 4:**
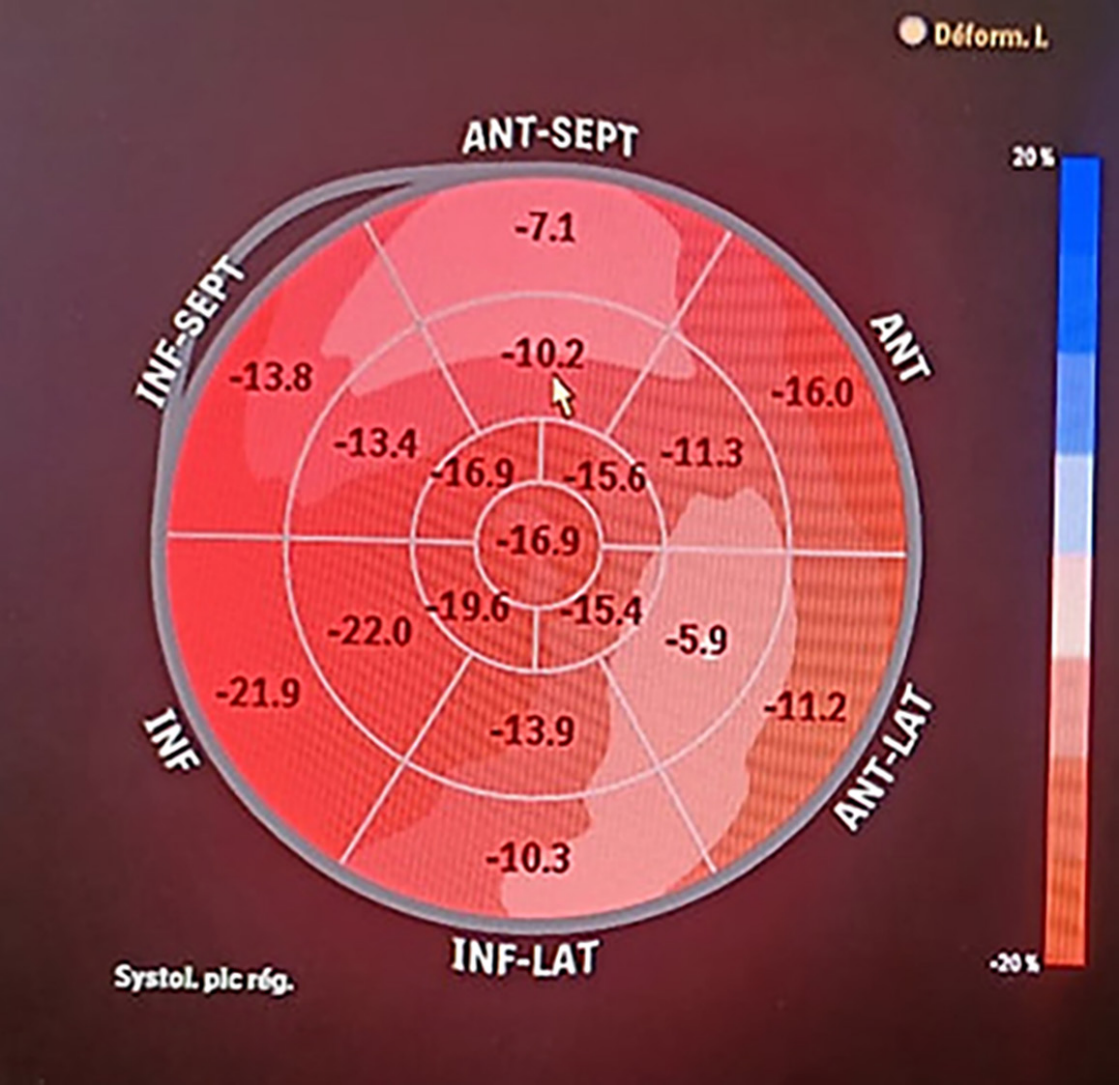
Bull's eyes of the peak systolic longitudinal strain after ASA procedure.

TTE and invasive control of pressure showed also a decrease in the LVOT gradient at 20 mm Hg (Figure 3). Two‐dimensional (2D) speckle‐tracking echocardiography demonstrated a reduced baseline global longitudinal strain (GLS) of −15.6% with dysfunction noted in the anteroseptal wall (Figure 4). There was not any mitral regurgitation after the ASA. The patient remained asymptomatic at 3 months of follow‐up and with persistence of a low LOVT gradient at TEE control at 15 mm Hg.

## 3 DISCUSSION

Congenital malformations of the LV papillary muscles, including the anomalous chordal insertion, is a rare anomaly leading to LVOT obstruction with an incidence of 1 per 26,000 echocardiograms in adults.^3^ HOCM could be associated with anomalous papillary muscle insertion (APMI) 13%–15%.^4^, ^5^, ^6^

Standard echocardiographic windows and modified windows should be used to identify the chordal abnormalities during the echocardiographic examination of patients with HCM.^7^

Symptomatic HOCM patients are usually treated first with β‐blockers or calcium channel inhibitors. In case of failure of medical treatment, physicians opted for surgery, no cases of ASA have been reported to our knowledge.^2^, ^5^, ^8^ Ogunmuyiwa et al.^2^ failed to treat a man with HOCM and abnormal chordae insertion, and they finished by doing myomectomy and chordae resection. The mechanism of obstruction is complex in such HOCM, sometimes we are unable to determine the underlying cause of obstruction.

Our case demonstrated that the main mechanism of obstruction in some patients is the hypertrophy of the basal part of the septum. In our patient, we decided to try to occlude the first septal with a balloon and to check whether the gradient will decrease during the procedure.

This test could be a solution to assess the immediate result of alcohol ablation. After this test, the operator could decide whether he continues the ASA procedure or opts for surgery.

In our case, the gradient had remarkably disappeared during the occlusion test and the ASA was performed with a good result and without any complications.

## 4 CONCLUSION

We showed that ASA is feasible in patients with anomalous chordae associated with HOCM. The analysis of imaging (MRI) and Doppler color is very important to decide. Moreover, the occlusion test with OTW could be very helpful if we obtained an immediate decrease in the outflow gradient.

## AUTHOR CONTRIBUTIONS

**Rania Hammami:** Supervision; writing – original draft. **Amine Kammoun:** Writing – original draft. **Majed Hassine:** Resources; validation; writing – review and editing. **Tarek Ellouze:** Writing – review and editing. **Rania Gargouri:** Writing – review and editing. **Leila Abid:** Writing – review and editing.

## FUNDING INFORMATION

None.

## CONFLICT OF INTEREST STATEMENT

The authors declare no conflict of interest.

## CONSENT

Written informed consent was obtained from the patient to publish this report in accordance with the journal's patient consent policy.

## Data Availability

None.
